# Supplementary material for: Threshold concentration and random collision determine the growth of the huntingtin inclusion from a stable core
Source: Commun Biol. 2021 Aug 16;4:971. doi: 10.1038/s42003-021-02460-z (PMC8368079; doi:10.1038/s42003-021-02460-z)
Supplement: Supplementary file 2 — Supplementary Information [file 42003_2021_2460_MOESM2_ESM.pdf]

Supplementary Information for

Threshold concentration and random collision determine the growth of the huntingtin inclusion from a stable core

Sen Pei, Theresa C. Swayne, Jeffrey F. Morris and Lesley Emtage*

*Correspondence to: lemtage@york.cuny.edu

This file includes:

Supplementary Movie Captions 1 and 2
Supplementary Notes 1-3
Supplementary Figures 1-11
Supplementary Tables 1-4
Supplementary References

Supplementary Movie 1. Inclusion body formed by mHtt(72Q)-GFP. An inclusion body and small particles of mHtt are seen to move in and around a single focal plane. The contrast has been adjusted to reveal the small particles. The movie was acquired at approximately 30 fps over 1 minute and the image is 16 μm wide.

Supplementary Movie 2. Inclusions formed by mHtt(72Q)ΔPRD-degron-GFP. Cluster-like inclusions are relatively immobile, but numerous small inclusions are seen to move around. The contrast has been adjusted to reveal the small inclusions. The movie was acquired of a single focal plane at approximately 30 fps over 30 seconds. The image is 21 μm wide.

Supplementary Note 1. The concentration threshold is dependent on polyQ tract length

Mutant Htt protein stability decreases with polyQ tract length[1–4]; a higher fraction of cytosolic mHttex1(103Q)-GFP should be unfolded compared to mHttex1(72Q)-GFP. Therefore, we predicted that at any particular cytoplasmic intensity, 103Q-expressing cells would have a higher burden of unfolded protein than 72Q-expressing cells, and that the threshold cytoplasmic intensity for inclusion formation should be lower for cells expressing longer polyQ tracts. To test this hypothesis, we assessed cytoplasmic mHttex1 intensity and inclusion formation for cells expressing mHttex1(103Q)-GFP ("103Q") using the same procedures as described above for mHttex1(72Q)-GFP ("72Q").

We began by comparing the frequency of inclusions in 103Q- and 72Q-expressing cells. We found that 45% of mid-log 72Q-expressing cells had an inclusion of some kind, while 70% of 103Q-expressing cells had one or more inclusions, a difference that was highly significant ($p=0.0017$ two-tailed t test assuming unequal variance; 4 independent replicates for each construct, $n=295$, 200 cells respectively). The majority (56%) of 103Q-expressing cells contained single, ovoid inclusions; furthermore, they demonstrated an increased propensity to form multiple inclusions or cluster-like inclusions, which together were found in 15% of cells expressing 103Q, compared to 3% of cells expressing 72Q (Supplementary Fig. 3). These results are consistent with our expectation that 103Q is more likely to unfold.

As with 72Q-expressing cells, we imaged 103Q-expressing cells over a period of 6-7 hours of growth. For 103Q-expressing cells that were born during the timecourse and then formed an inclusion body, the average time to form an inclusion was 1.6 ± 0.2 hours, compared to 1.6 ± 0.1 hours for 72Q-expressing cells (mean ± SEM, n=49, 56). However, both cytoplasmic GFP intensity and the intensity threshold for inclusion formation were markedly lower in 103Q-expressing cells.

While cytoplasmic intensities observed in 72Q-expressing cells ranged from 45 to 3194 AU, the cytoplasmic levels in 103Q-expressing cells ranged from 39 to 1024 AU, suggesting a selection against higher plasmid copy numbers, a large increase in Htt removal rate, or a combination of the two. Furthermore, we found that mutant Htt(103Q) is so unstable that 103Q-expressing cells will form an inclusion at very low cytoplasmic intensities: the apparent threshold for inclusion formation by 103Q was about 200 AU, compared to about 600 AU for 72Q (Supplementary Fig. 4). The shift to a lower threshold and the increase in inclusion frequency suggest that a higher percentage of cytoplasmic 103Q is unfolded compared to 72Q.

Supplementary Note 2. Inclusions lacking the characteristics of phase separation are insensitive to cytoplasmic Htt levels

Do other types of mHtt inclusion respond to an auxin-mediated reduction in cytoplasmic protein levels? To address this question, we used a mHtt construct that lacks the Htt proline-rich domain. HTT exon 1 encodes an N-terminal α-helical domain, the polyQ tract, and a proline-rich domain (PRD). The PRD has been shown to shown to directly interact with the htt17 α-helix and may form a proline helix[5,6].

Polyproline tracts have been shown to modulate the aggregation propensity of polyQ tracts, increasing the stability of polyQ sequences[7–11]. Consistent with the in vitro findings, deletion of the mHttex1 polyproline tract immediately after the polyQ tract has been found to dramatically alter inclusion number and size in *S. cerevisiae*[12,13]. Unlike inclusion bodies, ΔPRD inclusions form in cells with very low cytoplasmic mHttex1ΔPRD-GFP. Auxin-induced degradation using mHttex1ΔPRD-degron-GFP demonstrates that ΔPRD inclusions do not respond to changes in cytoplasmic concentration.

We expressed mHtt(72Q)ΔPRD-GFP ("ΔPRD") from the constitutive GPD promoter. Consistent with the original description, we saw excessive and abnormal aggregation of the ΔPRD construct. Instead of a single ovoid inclusion body, ΔPRD typically formed abundant small inclusions distributed throughout the cytoplasm. To test whether ΔPRD inclusions are sensitive to cytoplasmic levels of mHttex1ΔPRD, we introduced the degron sequence as described previously. Surprisingly, introduction of the degron sequence directly after the polyQ tract appeared to partially stabilize mHtt(72Q)ΔPRD, permitting it to accumulate to slightly higher levels in the cytoplasm and to form large, singular inclusions with fewer small distributed inclusions.

Despite the apparent partial stabilization of the protein, the large inclusions typically did not have the visible properties associated with phase separation found in inclusion bodies composed of mHtt(72Q)-GFP: they were frequently non-uniform in intensity and shape

(Supplementary Fig. 8a), and not as mobile (Supplementary Movie 1, 2). Cytoplasmic mHttex1ΔPRD-degron-GFP levels were still robustly reduced in comparison to mHttex1-GFP. Nevertheless, as some cells retained visible cytoplasmic GFP, we were able to determine whether the ΔPRD inclusions would respond to AID.

The mHttex1(72Q)ΔPRD-degron-GFP construct was introduced into cells expressing Tir1. Incubation of mHttex1ΔPRD-GFP in 250 μM NAA did reduce cytoplasmic levels in a subset of cells, although the cytoplasmic ΔPRD levels showed no response to auxin in most cells (Supplementary Fig. 8a). Time-lapse imaging of non-responding cells revealed that the cytoplasmic Httex1ΔPRD-GFP is incorporated into numerous small aggregates (Supplementary Movie 2), which are unlikely to contain much soluble protein, based on previous biochemical characterization[12,13]. However, the appearance of the cytoplasmic mHttex1ΔPRD-GFP was diffuse in some cells, and levels did drop in response to auxin. We measured the change in inclusion size in the subset of cells that showed reduced cytoplasmic mHttex1ΔPRD-degron-GFP intensity in response to NAA. We found that the reduction in cytoplasmic intensity had no measurable effect on inclusions; there was little change in appearance or size (Supplementary Fig. 8b).

Supplementary Note 3. Inclusion body growth characteristics do not depend on polyQ tract length

We examined the growth rate of inclusion bodies formed from mHttex1(103Q)-GFP. The 103Q cells remained healthy and cells continued to be born during the full timecourse (Supplementary Fig. 11a). The rate of growth of 103Q inclusion bodies, as with 72Q-expressing cells, was dependent on cytoplasmic mHtt levels (Supplementary Fig. 11b). The growth followed the same trajectory observed for inclusion bodies formed by 72Q: considering inclusions with diameters of approx. 400 nm and above and cytoplasmic intensities ≥ 450 AU, we see that the change in radius is linear with time, while the change in volume is proportional to t^3 (Supplementary Fig. 11c). Consistent with the proposition that a higher fraction of the cytoplasmic protein unfolds, the growth of 103Q inclusion bodies in cells with cytoplasmic intensities between 500-1000 is much faster than that of 72Q inclusions in cells of similar intensity (Figure 8a, Supplementary Fig. 11c).

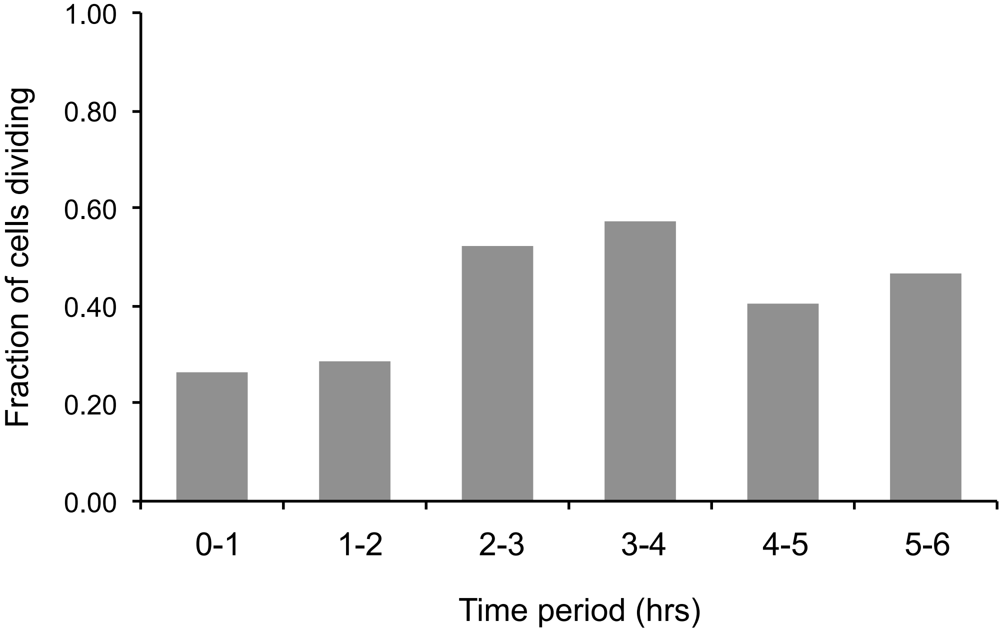

Supplementary Figure 1. Cells continue to divide throughout the imaging session. For the 42 initial cells, the percentage of cells dividing within the indicated time period is shown.

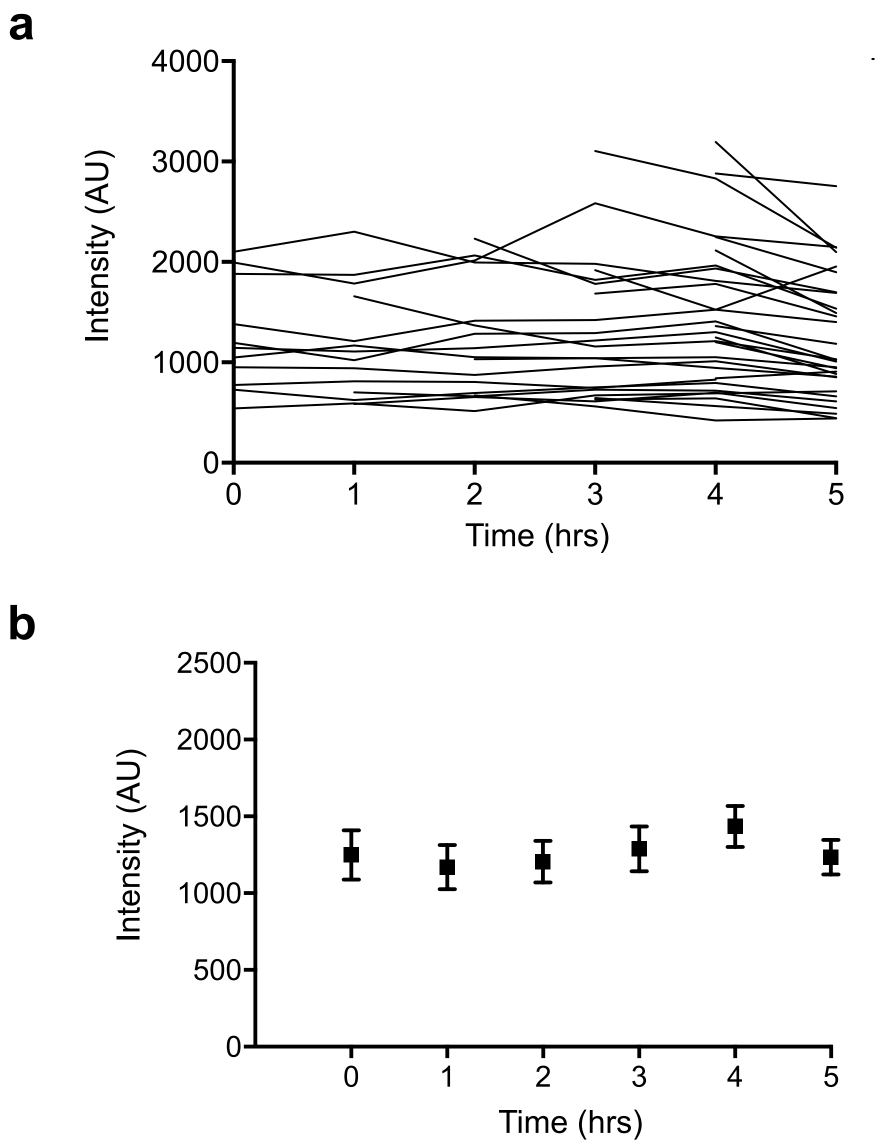

Supplementary Figure 2. Cytoplasmic mHtt-GFP intensity is stable over 5 hours of imaging. a, Cytoplasmic intensities over time for 31 randomly selected inclusion body-forming cells are shown. Traces beginning after t = 0 represent cells that were born during the course of the experiment. **b,** Mean intensity ± SEM is shown for the same 31 inclusion body-forming cells.

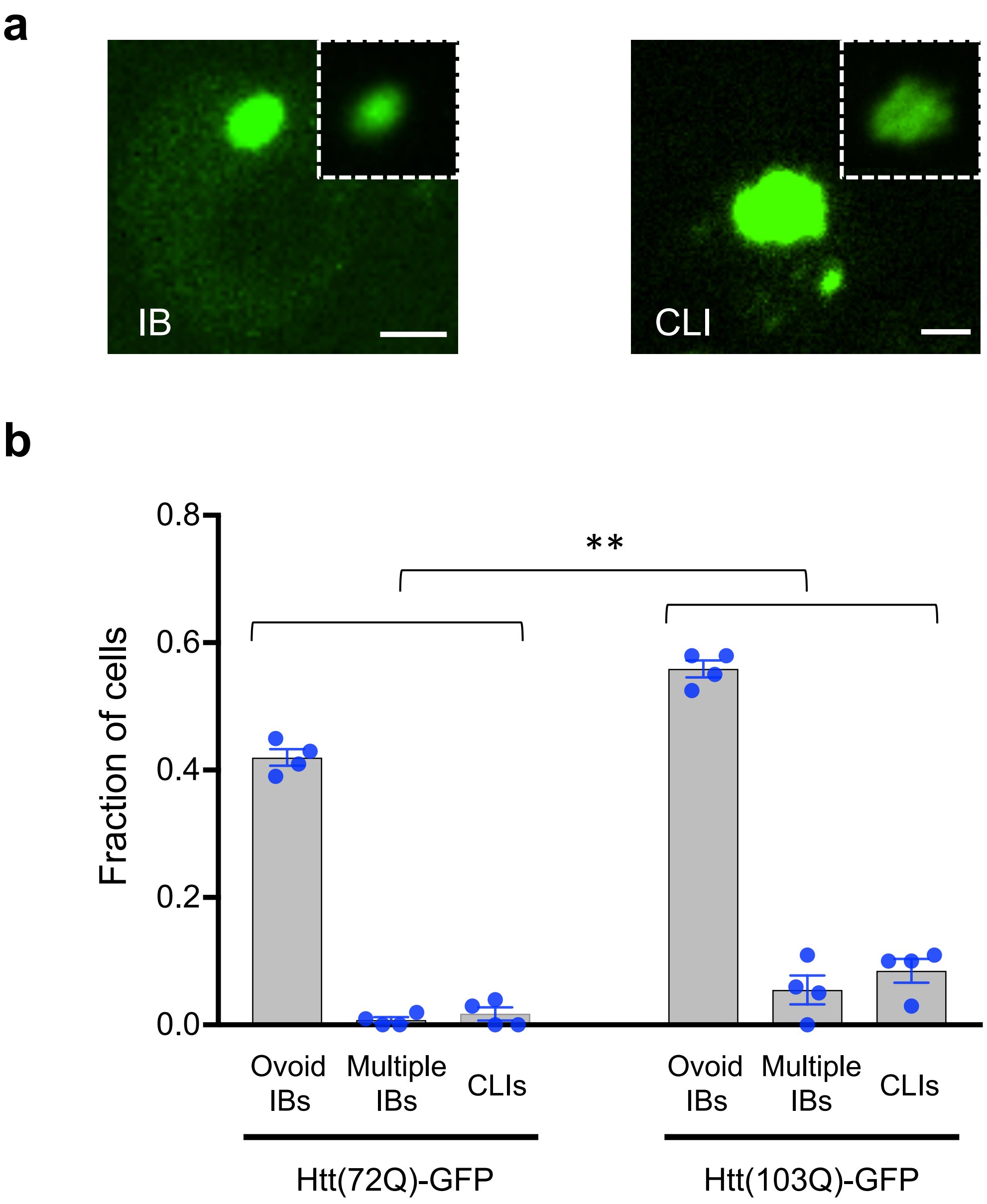

Supplementary Figure 3. mHtt(103Q)-GFP has a higher propensity to form inclusions than mHtt(72Q)-GFP. **a,** Each inclusion was categorized as an inclusion body (IB) or a cluster-like inclusion (CLI). The inset is a contrast-adjusted image of the inclusion to show the uneven distribution of material in the CLI. Scale bar, 1 μm. **b,** The fraction of cells containing a single ovoid IB, multiple IBs or cluster-like inclusions is shown for mHttex1(103Q)-GFP and mHttex1(72Q)-GFP-expressing cells. Bars indicate mean ± SEM. Overall, 45% of mid-log 72Q-expressing cells had an inclusion of some kind, while 70% of 103Q-expressing cells had one or more inclusions, a highly significant difference (p=0.0017, two-tailed t test assuming unequal variance; 4 independent replicates each, n=295, 200 cells respectively).

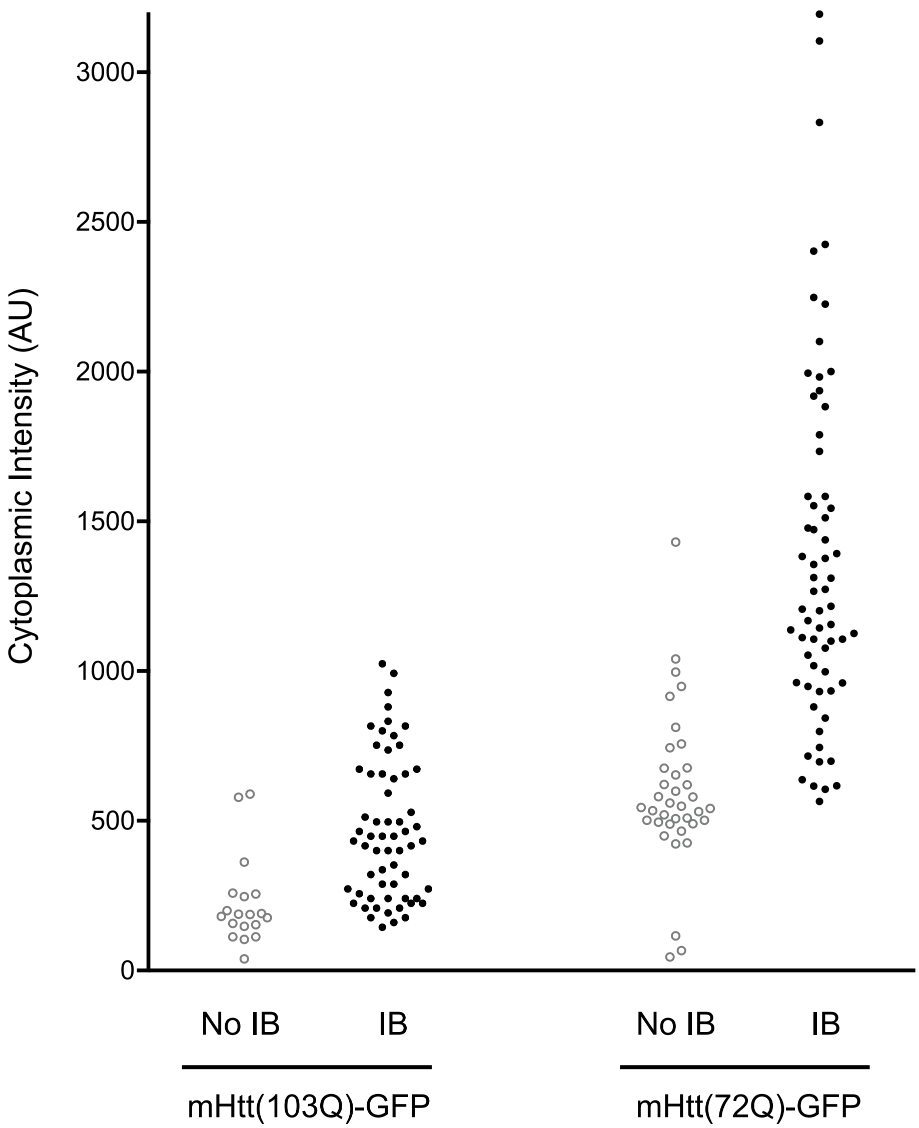

Supplementary Figure 4. Lowered threshold for inclusion body formation in mHtt(103Q)-GFP-expressing cells. The cytoplasmic intensity of mHttex1(103Q)-expressing cells that fail to form, or form, inclusion bodies (IBs) during a 7-hour timecourse (n = 20 and 61, respectively). To facilitate comparison, the data for mHttex1(72Q)-GFP-expressing cells has been included (Figure 3c).

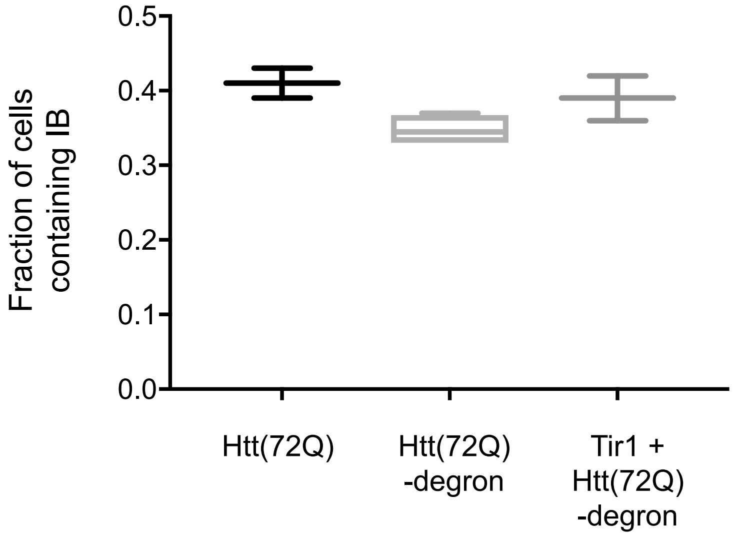

Supplementary Figure 5. The insertion of a degron between mHttex1 and GFP does not significantly alter protein stability in the absence of auxin. Inclusion body (IB) frequency was determined in cells expressing mHttex1(72Q) fused to GFP with or without the degron sequence. IB formation was also measured in cells co-expressing mHtt-degron-GFP and the E3 ligase Tir1. There was no significant difference in IB frequency between the cells expressing mHttex1(72Q) with and without the degron before GFP, nor a difference between mHtt-degron-GFP with and without co-expression of Tir1 ($p=0.14$, 0.37 respectively, two-tailed t test assuming unequal variance; $n = 133$, 272, 130 cells in the order presented). Whiskers indicate minimum and maximum, the mean of 2-4 individual imaging sessions is shown.

a

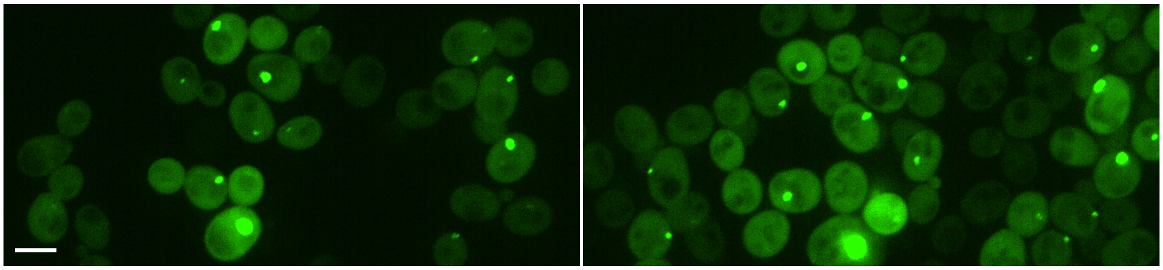

b

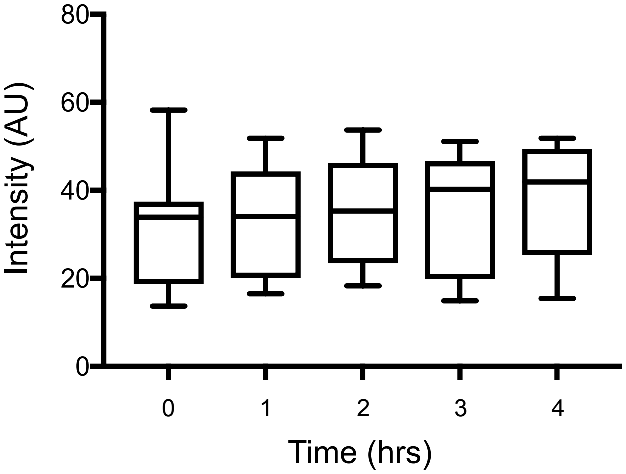

Supplementary Figure 6. Vehicle-treated cells expressing Tir1 and mHtt-degron-GFP show no change in cytoplasmic intensity. a, Cells grown identically to those in Figure 4 were treated with the vehicle (95% ethanol) and imaged every 15 minutes for 4 hours. A representative field of cells at the beginning (0 hrs) and end (4 hrs) of the timecourse. Scale bar, 4 μm. **b,** Average cytoplasmic intensity of 11 randomly sampled cells (whiskers indicate minimum and maximum values).

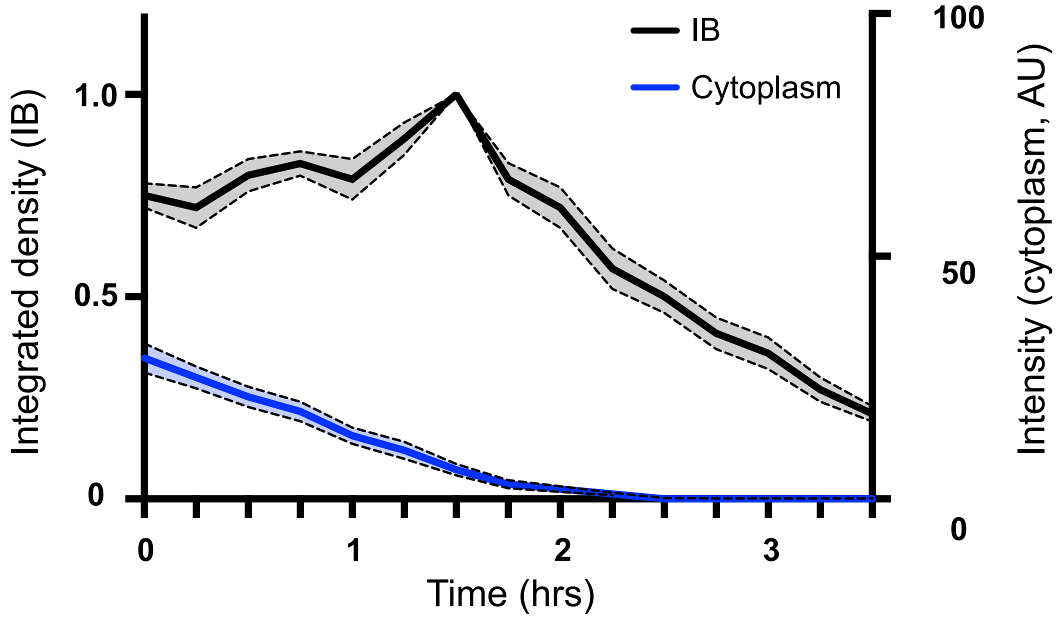

Supplementary Figure 7. Mutant Htt inclusion bodies decrease in integrated density when cytoplasmic mHtt levels drop to low levels. Normalized integrated density of inclusion body (IB, left axis) and cytoplasmic intensity (right axis) for 19 auxin-treated cells, showing mean (solid line) ± SEM (dotted lines). Intensity traces were corrected for bleaching and aligned in time to the maximum intensity.

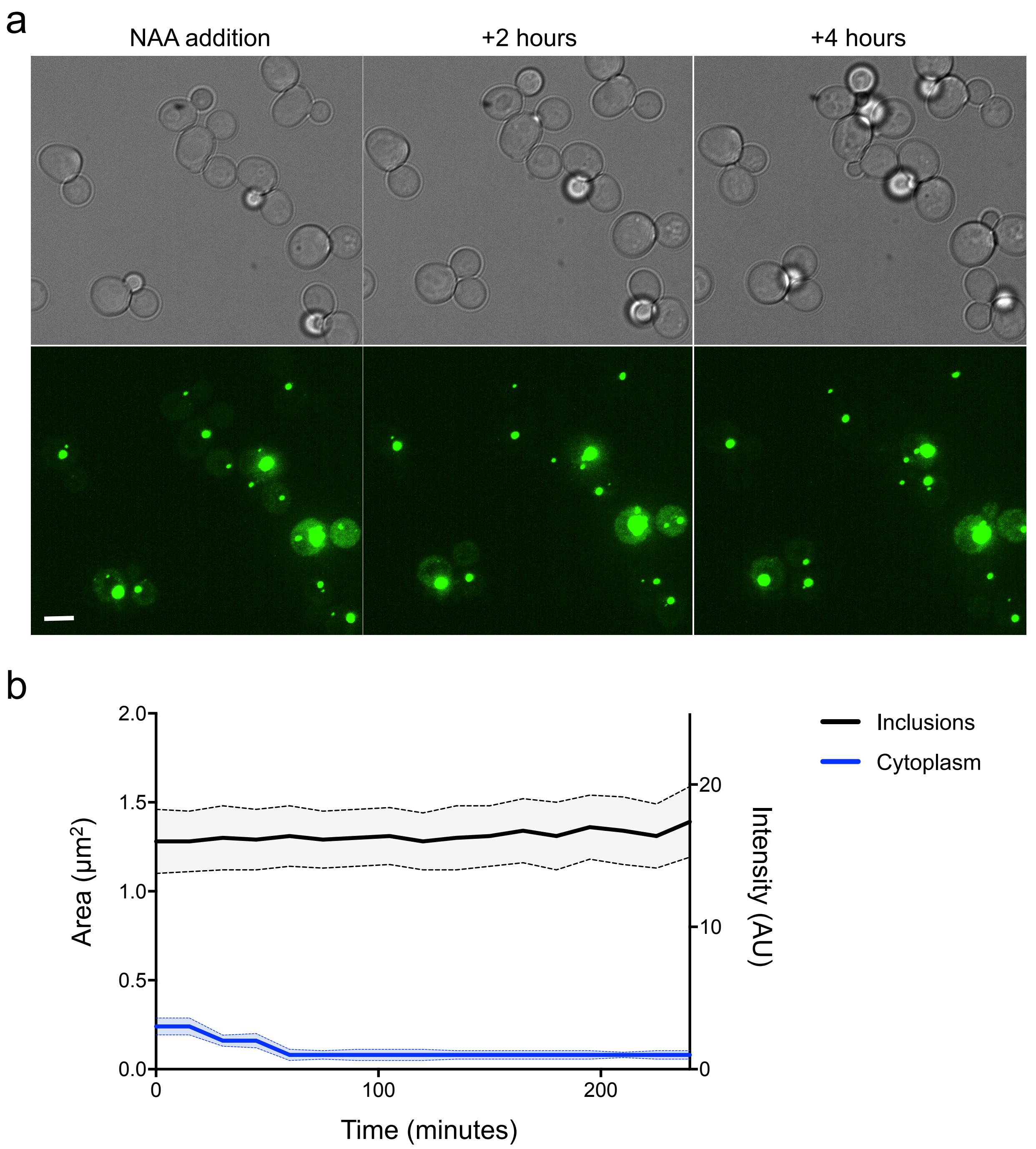

Supplementary Figure 8. Mutant HttΔPRD inclusions do not respond to reductions in cytoplasmic mHttΔPRD. a, Maximum projection of cells expressing mHtt(72Q)ΔPRD-degron-GFP in brightfield (top) and fluorescence (bottom), showing reduction in cytoplasmic ΔPRD levels in some cells after incubation with 250 μM NAA. Scale bar, 5 μm. **b,** For 17 cells with decreased cytoplasmic ΔPRD in response to auxin, the area of the inclusions was measured every 15 minutes for 4 hours. The average area and cytoplasmic intensities are shown (shaded area indicates SEM).

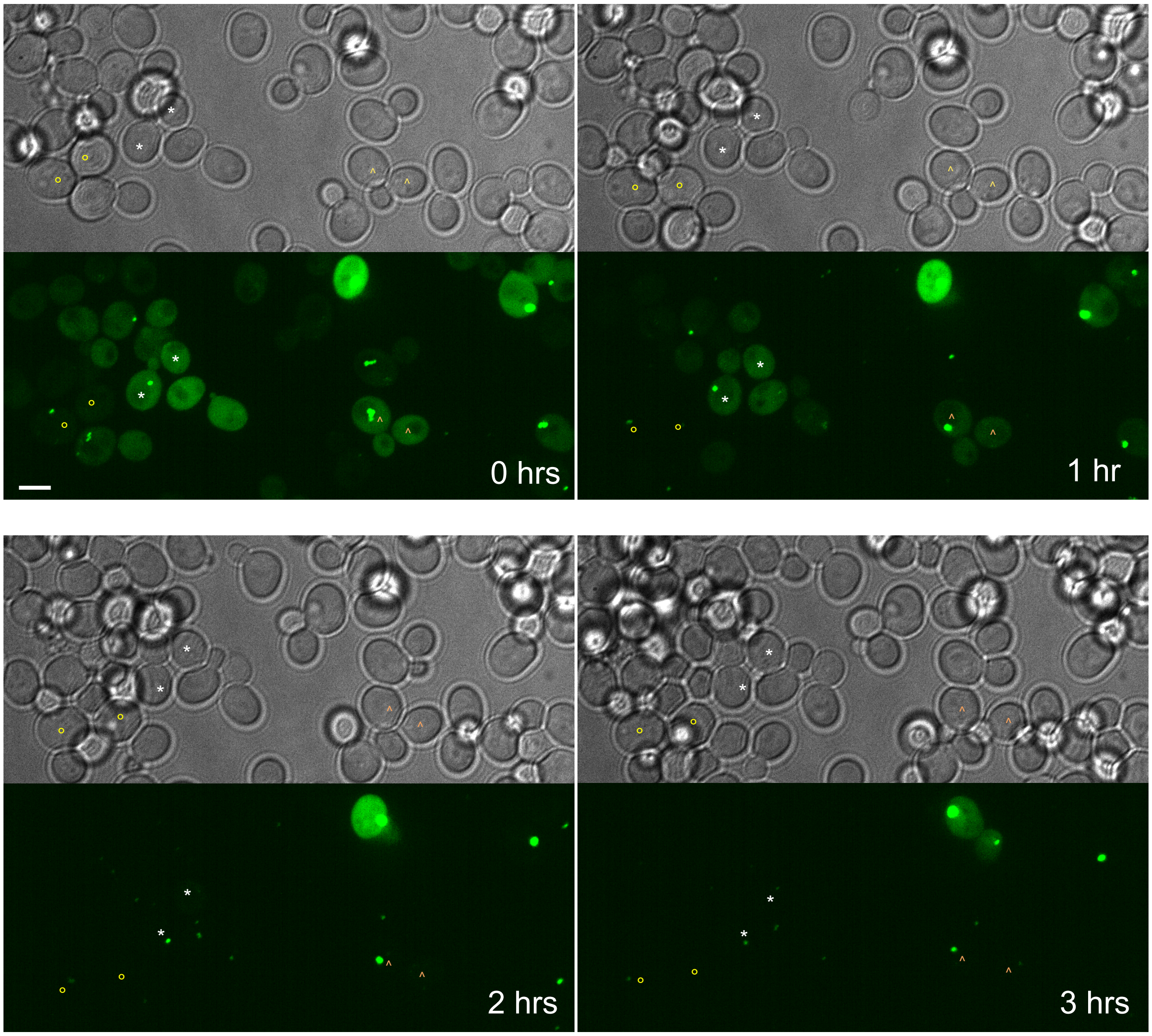

Supplementary Figure 9. Brightfield and fluorescent images of a field of mHtt-degron-GFP-expressing cells exposed to auxin. A field of cells containing the 6 cells shown in Figure 6 is shown; maximum-intensity projections of confocal images (lower panels) with a brightfield image of the same field (upper panels). The cells shown in Figure 7 are indicated with the symbols °(yellow, left-hand column in Fig. 7), *(white, central column in Fig. 7), ^(orange, right-hand column in Fig. 7). Scale bar, 4 μm.

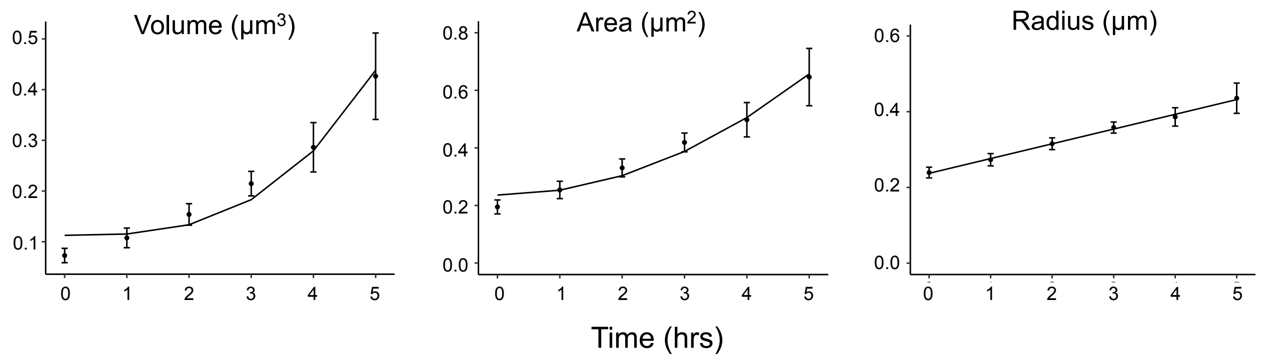

Supplementary Figure 10. Growth rate for all mHttex1(72Q)-expressing inclusion bodies. Average inclusion body volume, area and radius were plotted over time for all 25 IBs (error bars indicate SEM). Best-fit curves to functions of t^3 for volume (R^2 = 0.95), t^2 for area (R^2 = 0.97) and t for radius (R^2 = 0.995) are shown.

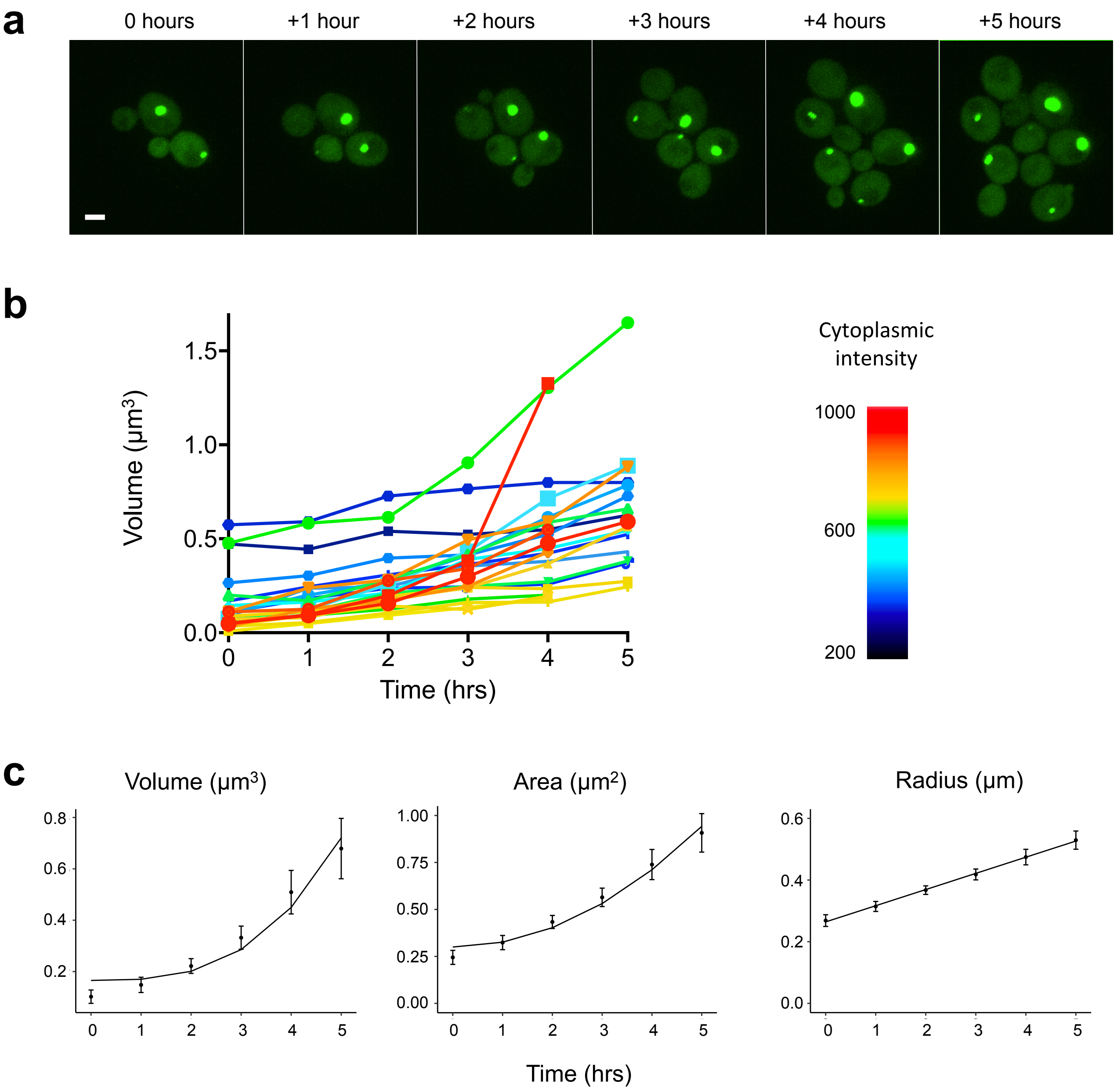

Supplementary Figure 11. Htt(103Q)-GFP inclusion body growth rate for radius is linear with time. a, A typical field of cells imaged over a 5 hour timecourse is shown; the maximum projection includes those sections containing inclusion bodies (IBs). The contrast has been set to identical parameters for all images. Scale bar, 2 μm. **b,** The measured volume of 23 IBs over 3-5 hours. The growth curves were aligned to time of first observation of each IB. The color of each IB trace indicates the average cytoplasmic intensity of the cell containing the IB (bar, right). **c,** Average IB volume, area and radius are plotted over time for the 17 IBs in cells with cytoplasmic intensities >450 AU (error bars indicate SEM). Fits of the growth of volume with t^3 (R^2 = 0.94), area with t^2 (R^2 = 0.98) and radius with t (R^2 = 0.999) are shown.

Quantity linear with time	Curve fit for radius vs		Curve fit for area vs		Curve fit for volume vs	
	t^n	**R^2**	***t^n***	**R^2**	***t^n***	**R^2**
**Radius**	t	0.99	t^2	0.99	t^3	0.97
**Area**	$t^{1/2}$	0.84	t	0.95	$t^{3/2}$	0.98
**Volume**	$t^{1/3}$	0.69	$t^{2/3}$	0.85	t	0.90

Supplementary Table 1. Curves fits for growth of inclusion body with time. For each model, linear growth of inclusion body (IB) radius with time, linear growth of area with time, and linear growth of volume with time, curves were fitted to plots of radius, area and volume. For each model, the prediction of linear growth was extended to the other IB parameters: for example, if the growth of the radius with time is linear, it follows that the growth of area will grow as t^2, and volume will grow as t^3. Coefficients of determination (R^2) for all curve fits of IB volume, area and radius with t are shown. Data for IBs in cells with cytoplasmic intensity >1100 was used (n=16).

Cytoplasmic Intensity	Slope of radius vs. time (µm/hour ± SE)	95% confidence interval for slope	R^2 For line fit
2695	0.071 ± 0.009	0.048 to 0.094	0.93
2654	0.075 ± 0.006	0.060 to 0.091	0.98
2181	0.052 ± 0.004	0.036 to 0.069	0.99
2130	0.061 ± 0.01	0.003 to 0.12	0.91
2087	0.060 ± 0.007	0.031 to 0.089	0.98
2010	0.047 ± 0.01	0.001 to 0.093	0.91
1981	0.038 ± 0.02	-0.033 to 0.11	0.73
1857	0.039 ± 0.007	0.009 to 0.068	0.94
1782	0.062 ± 0.02	0.013 to 0.11	0.85
1686	0.054 ± 0.005	0.040 to 0.069	0.96
1616	0.034 ± 0.003	0.024 to 0.043	0.96
1445	0.041 ± 0.003	0.033 to 0.050	0.97
1392	0.044 ± 0.007	0.016 to 0.073	0.96
1329	0.029 ± 0.006	0.01 to 0.049	0.89
1272	0.021 ± 0.003	0.014 to 0.029	0.91
1234	0.049 ± 0.01	0.014 to 0.085	0.87
1091	0.025 ± 0.006	0.007 to 0.043	0.79
1058	0.020 ± 0.02	-0.082 to 0.12	0.26
1029	0.027 ± 0.002	0.022 to 0.032	0.97
979	0.040 ± 0.008	0.013 to 0.067	0.88
859	0.021 ± 0.003	0.012 to 0.030	0.88
782	0.017 ± 0.008	-0.008 to 0.042	0.61
743	0.022 ± 0.004	0.010 to 0.034	0.87
687	0.026 ± 0.01	-0.011 to 0.063	0.62
504	0.0084 ± 0.005	-0.0053 to 0.022	0.33

Supplementary Table 2. Slope of line fitted to a plot of radius vs time for individual inclusion bodies. The cytoplasmic intensity of the cell in which the inclusion body is found is given in the first column, the slope of the fitted line is given in the second column (best-fit ± SE). Goodness of fit is indicated by the 95% confidence interval and the coefficient of determination, R^2.

Plasmid	Description	Source
pEB4	Htt(72Q)-GFP/CEN/LEU2, derived from p415GPD	14
pEB6	Htt(72Q)ΔPRD-GFP/CEN/LEU2, derived from p415GPD	This study
pEB11	Htt(103Q)-GFP/CEN/LEU2, derived from p415GPD	This study
pEB18	Htt(72Q)-IAA$^{71-114}$-GFP/CEN/LEU, derived from p415GPD	This study
pEB28	Htt(72Q)ΔPRD-IAA$^{71-114}$-GFP/CEN/LEU2, derived from p415GPD	This study
OsTir1	HO-Tir1	15
GCN5-mCh	GCN5-mCherry- IAA$^{71-114}$	15

Supplementary Table 3. A list of plasmids used in this study

Strain	*Genotype*	*Source*
mHtt(72Q)-GFP	*MATa his3Δ1 leu2Δ0 met15Δ0 ura3Δ0 pEB4 [pGDP-mHtt(72Q)-GFP::LEU2]*	Aktar et al., 2019
mHtt(72Q)-degron-GFP	*MATa his3Δ1 leu2Δ0 met15Δ0 ura3Δ0 pEB18 [pGDP-mHtt(72Q)-degron-GFP::LEU2]*	This study
mHtt(103Q)-GFP	*MATa his3Δ1 leu2Δ0 met15Δ0 ura3Δ0 pEB11 [pGDP-mHtt(103Q)-GFP::LEU2]*	This study
mHtt(72Q)ΔPro-GFP	*MATa his3Δ1 leu2Δ0 met15Δ0 ura3Δ0 pEB6 [pGDP-mHtt(72Q)ΔPro-GFP::LEU2]*	This study
HO-TIR1	*MATa his3Δ1 leu2Δ0 met15Δ0 ura3Δ00 HO-TIR::kanMx*	This study
HO-TIR1 mHtt(72Q)-degron-GFP	*MATa his3Δ1 leu2Δ0 met15Δ0 ura3Δ00 HO-TIR1::KanMx pEB18 [pGDP-mHtt(72Q)-degron-GFP::LEU2]*	This study
HO-TIR1 mHtt(72Q)ΔPRD-degron-GFP	*MATa his3Δ1 leu2Δ0 met15Δ0 ura3Δ00 HO-TIR1::KanMx pEB28 [pGDP-mHtt(72Q)ΔPRD-degron-GFP::LEU2]*	This study

Supplementary Table 4. A list of strains used in this study.

Supplementary References

1. Chen, S., Berthelier, V., Yang, W. & Wetzel, R. Polyglutamine aggregation behavior in vitro supports a recruitment mechanism of cytotoxicity. *J. Mol. Biol.* **311**, 173–182 (2001).
2. Fiumara, F., Fioriti, L., Kandel, E. R. & Hendrickson, W. A. Essential role of coiled coils for aggregation and activity of Q/N-rich prions and PolyQ proteins. *Cell* **143**, 1121–1135 (2010).
3. Scherzinger, E. *et al.* Huntingtin-encoded polyglutamine expansions form amyloid-like protein aggregates in vitro and in vivo. *Cell* **90**, 549–558 (1997).
4. Slepko, N. *et al.* Normal-repeat-length polyglutamine peptides accelerate aggregation nucleation and cytotoxicity of expanded polyglutamine proteins. *Proc. Natl. Acad. Sci. U. S. A.* **103**, 14367–14372 (2006).
5. Kim, M. W., Chelliah, Y., Kim, S. W., Otwinowski, Z. & Bezprozvanny, I. Secondary structure of Huntingtin amino-terminal region. *Struct. Lond. Engl. 1993* **17**, 1205–1212 (2009).
6. Warner, J. B. *et al.* Monomeric Huntingtin Exon 1 Has Similar Overall Structural Features for Wild-Type and Pathological Polyglutamine Lengths. *J. Am. Chem. Soc.* **139**, 14456–14469 (2017).
7. Bhattacharyya, A. *et al.* Oligoproline Effects on Polyglutamine Conformation and Aggregation. *J. Mol. Biol.* **355**, 524–535 (2006).
8. Darnell, G., Orgel, J. P. R. O., Pahl, R. & Meredith, S. C. Flanking polyproline sequences inhibit beta-sheet structure in polyglutamine segments by inducing PPII-like helix structure. *J. Mol. Biol.* **374**, 688–704 (2007).

9. Darnell, G. D., Derryberry, J., Kurutz, J. W. & Meredith, S. C. Mechanism of Cis-Inhibition of PolyQ Fibrillation by PolyP: PPII Oligomers and the Hydrophobic Effect. *Biophys. J.* **97**, 2295–2305 (2009).

10. Falk, A. S. *et al.* Structural Model of the Proline-Rich Domain of Huntingtin Exon-1 Fibrils. *Biophys. J.* **119**, 2019–2028 (2020).

11. Posey, A. E. *et al.* Profilin reduces aggregation and phase separation of huntingtin N-terminal fragments by preferentially binding to soluble monomers and oligomers. *J. Biol. Chem.* **293**, 3734–3746 (2018).

12. Dehay, B. & Bertolotti, A. Critical role of the proline-rich region in Huntingtin for aggregation and cytotoxicity in yeast. *J. Biol. Chem.* **281**, 35608–35615 (2006).

13. Gruber, A. *et al.* Molecular and structural architecture of polyQ aggregates in yeast. *Proc. Natl. Acad. Sci.* **115**, E3446–E3453 (2018).

14. Aktar, F. *et al.* The huntingtin inclusion is a dynamic phase-separated compartment. *Life Sci. Alliance* **2**, (2019).

15. Papagiannakis, A., de Jonge, J. J., Zhang, Z. & Heinemann, M. Quantitative characterization of the auxin-inducible degron: a guide for dynamic protein depletion in single yeast cells. *Sci. Rep.* **7**, 4704 (2017).
