# Supplementary material for: Threshold concentration and random collision determine the growth of the huntingtin inclusion from a stable core
Source: Commun Biol. 2021 Aug 16;4:971. doi: 10.1038/s42003-021-02460-z (PMC8368079; doi:10.1038/s42003-021-02460-z)
Supplement: Supplementary file 3 — Description of Additional Supplementary Files [file 42003_2021_2460_MOESM3_ESM.pdf]

Description of Additional Supplementary Files

File Name: Supplementary Movies 1 and 2

Description: *A comparison of inclusions formed by mHtt(72Q)-GFP (Movie 1) and mHtt(72Q)ΔPRD-degron-GFP (Movie 2).* The movies were acquired at approximately 30 fps; Movie 1 was collected over 1 minute, and Movie 2 was collected over 30 seconds. The image is 15 µm wide in Movie 1 and 18 µm wide in Movie 2.

File Name: Supplementary Data 1

Description: Source data for Figure 2-9.
