# Supplementary figures and images for: Threshold concentration and random collision determine the growth of the huntingtin inclusion from a stable core
Source: Commun Biol. 2021 Aug 16;4:971. doi: 10.1038/s42003-021-02460-z (PMC8368079; doi:10.1038/s42003-021-02460-z)

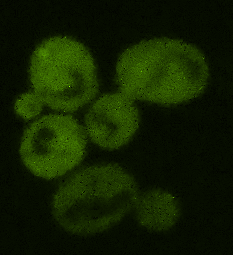

Supplement: Supplementary file 4 — Supplementary Movie 1 [file 42003_2021_2460_MOESM4_ESM.gif]
